# ID 72 - Estratégias de Divulgação do Conhecimento: democratizando a Avaliação de Tecnologias em Saúde em um hospital universitário federal

**DOI:** 10.5327/2237-9622.2025.v34s1.72

**Published:** 2025-11-25

**Authors:** Ellen Daiane Biavatti de Oliveira Algeri, Sousa Ellen Daiane Biavatti de Oliveira, Anderson Luis Mota Sampaio, Dayse Sanches Guimarães Paião, Alexandre Teixeira Andrade, Iara Beatriz Andrade de Sousa

## Abstract

**Introdução:**

Com o objetivo de democratizar a Avaliação de Tecnologias em Saúde (ATS) no âmbito no Hospital Universitário da Universidade Federal da Grande Dourados (HU-UFGD) filial Ebserh, o Núcleo de Avaliação de Tecnologias em Saúde (Nats) adotou algumas estratégias: a) Episódios em formato de quadrinhos intitulado: "Conheça o Nats". O quadrinho é produzido pela coordenação do núcleo com uma linguagem atual e acessível trazendo informações estratégicas sobre o Nats de uma maneira mais leve e descontraída com vistas a instigar a comunidade hospitalar a respeito da atuação do Nats; b) Animação em formato de vídeo curto: a animação foi produzida como outra estratégia de democratizar a ATS com objetivo de alcançar aquelas pessoas que possuem maior facilidade de aprendizado com a comunicação oral. As duas estratégias são divulgadas via e-mail institucional e apresentados no Painel Digital/Televisão Corporativa da Unidade de Comunicação, localizada na entrada de funcionários; c) disponibilização dos pareceres do Nats na íntegra no Catálogo de Sistemas do HU nomeado "Produtos de ATS". O Catálogo é uma ferramenta para facilitar o acesso aos inúmeros sistemas e serviços necessários aos colaboradores do HU-UFGD/Ebserh para realizarem suas atividades laborais, por meio da centralização em um catálogo de links de sistemas, com interface simples e intuitiva. Quando o colaborador clica no navegador, automaticamente ele é direcionado para o catálogo de sistema que possui excelente adesão e acesso restrito no ambiente hospitalar; d) criação pelos membros do Nats de um Painel em Power BI. Ele apresenta o cenário da ATS no ambiente hospitalar de uma maneira dinâmica e interativa; permite o monitoramento em tempo real e fornece uma visão consolidada das principais métricas. O painel possibilitou a organização e a visualização dos dados de maneira coesa, facilitando a análise e a compreensão do status atual da ATS na instituição pelos colaboradores e pela gestão. O painel traz informações como: documentos concluídos e em andamento; médias de tempo de execução e horas dispensadas para elaboração dos documentos técnicos, tempo de resposta (em dias), tanto no geral quanto por tipo de documento elaborado; tipo de tecnologia avaliada e links de acesso rápido aos pareceres, orientações de como solicitar uma ATS; regimento interno; membros do Nats; documentos e formulários e; acesso a diversos sites e materiais de interesse.

**Método:**

A apresentação ocorrerá de forma oral com auxílio de recursos audiovisuais como: datashow, computador com acesso à internet e equipamento de som. Inicialmente, será apresentado os episódios em formato de quadrinhos: "Conheça o Nats" seguido da animação/vídeo de divulgação. Na sequência, serão apresentadas as imagens do Catálogo de Sistemas e acessos as Abas relacionadas ao Nats. A exposição oral será finalizada com a apresentação do Painel em Power BI.

